# Anticancer Secondary Metabolites: From Ethnopharmacology and Identification in Native Complexes to Biotechnological Studies in Species of Genus *Astragalus* L. and *Gloriosa* L.

**DOI:** 10.3390/cimb44090267

**Published:** 2022-08-26

**Authors:** Iliana Ionkova, Aleksandar Shkondrov, Yancho Zarev, Ekaterina Kozuharova, Ilina Krasteva

**Affiliations:** Department of Pharmacognosy, Faculty of Pharmacy, Medical University of Sofia, 2 Dunav Str., 1000 Sofia, Bulgaria

**Keywords:** plant anticancer compounds, in vitro production, saponins, flavonoids, alkaloids, *Astragalus*, *Gloriosa*, *ethnobotany*, *conservation*, *Agrobacterium rhizogenes*

## Abstract

Some of the most effective anticancer compounds are still derived from plants since the chemical synthesis of chiral molecules is not economically efficient. Rapid discovery of lead compounds with pronounced biological activity is essential for the successful development of novel drug candidates. This work aims to present the chemical diversity of antitumor bioactive compounds and biotechnological approaches as alternative production and sustainable plant biodiversity conservation. *Astragalus* spp., (Fabaceae) and *Gloriosa* spp. (Liliaceae) are selected as research objects within this review because they are known for their anticancer activity, because they represent two of the largest families respectively in dicots and monocots, and also because many of the medicinally important plants are rare and endangered. We summarized the ethnobotanical data concerning their anticancer application, highlighted the diversity of their secondary metabolites possessing anticancer properties such as saponins, flavonoids, and alkaloids, and revealed the potential of the in vitro cultures as an alternative way of their production. Since the natural supply is limited, it is important to explore the possibility of employing plant cell or organ in vitro cultures for the biotechnological production of these compounds as an alternative.

## 1. Introduction

Malignant diseases are the second cause of mortality, and their treatment remains a serious problem [1]. Some of the most powerful products in cancer therapy are still obtained from plants because the chemical synthesis of these chiral molecules is not economical [2,3]. Identification of plants that are efficient in cancer treatment relates to ethnobotanical and ethnopharmacological records; however, finding such information is not easy. The origin of the word “cancer” is credited to the Greek physician Hippocrates (460–370 BC), but the oldest description of this disease dates back to about 3000 BC in Egypt [1]. Cancer is not a modern disease, but research shows that it was only about a third as common in medieval Britain as in modern Britain [4]. Up to 50% prevalence of cancer is recorded at the time of death in modern Britain, which is explained by the effects of modern carcinogens, the spread of viruses that trigger malignancy, industrial pollutants, etc. [5]. Although cancer has been known since ancient times and has long been studied, neither the tumor types nor their causes are clear and well defined [6]. This is a serious challenge to the ethnobotanical research of cancer treatment, but at the same time, the results are rewarding. Plant-specific secondary metabolites have long been seen as a prospective approach in human therapy [7]. The interest in plant secondary metabolites from research to industry increases because synthetic chemicals are perceived as potentially toxic [8]. Many compounds are difficult to be synthesized via chemosynthesis, or the cost of their synthesis outweighs their commercial availability [9,10]. Most of the plant secondary metabolites with pharmaceutical use are still isolated from wild or cultivated plants. Plant resources, however, are not endless. Many of these plant species are endangered either due to their excessive collection or their limited distribution. Sometimes there are conservational restrictions, which can limit the commercial production of some compounds from wild populations. Some valuable substances can only be isolated from extremely rare plants. Conservation of biodiversity is curtailed [11,12,13,14,15]. A good and comparatively inexpensive method to obtain plant biomass for sufficient bioactive compounds’ extraction is cultivation [16,17,18], although, for some plants, it is difficult or takes several years. Additionally, isolating pharmaceutical products from plants is difficult due to their extremely low concentrations. However, modern science and practice have found a solution for these complications. It has become possible to use plant cells to produce specific pharmaceutical products by applying biotechnological approaches. The biotechnological approach offers a quick and efficient method of producing highly valuable compounds [19,20,21]. In this context, alternative methods for producing secondary metabolites appear as plant cell and tissue culture techniques. Starting from callus tissue, cell suspension cultures can be established and can even be grown in large bioreactors. In addition, the biotechnological production of these plant compounds is more environmentally friendly. Some of the advantages of the in vitro techniques are the propagation of the plants in aseptic controlled conditions and their large-scale production in a year-round system without seasonal constraints [22]. The plant cell techniques provide some highly efficient methods for isolating and extracting the secondary metabolites within a short time compared to the wild plant populations. The simplicity of these in vitro-produced tissue methods makes them suitable for commercial application [23]. Additionally, some metabolites can be produced by in vitro cultures, but generally, they are not found in intact plants [24]. 

In this work, we aim to integrate the chemical diversity of antitumor bioactive compounds and medicinal plant biodiversity conservation with a biotechnological approach. The research objects *Astragalus* spp. div., (Fabaceae) and *Gloriosa* spp. div. (Liliaceae) were selected firstly because they are known for their anticancer activity, secondly because they represent two of the largest families respectively in dicots and monocots, and also because many of the medicinally important plants are rare and endangered. The aim of this review study is (1) to summarise the ethnobotanical data about their anticancer application, (2) to highlight the diversity of their secondary metabolites possessing anticancer properties, (3) to reveal the potential of the in vitro cultures as an alternative way of their production.

## 2. Materials and Methods

We accessed Web of Science (https://www.webofscience.com/wos/woscc/basic-search, accessed on 1 January 2022) and PubMed (https://pubmed.ncbi.nlm.nih.gov/advanced/, accessed on 1 January 2022). A time range of 2018–2022 was set. The following keywords were used: “*Astragalus*”, “*Gloriosa”*, “traditional”, “ethnobotany”, “cancer”, “secondary metabolites”, “biotechnology”, and “tissue cultures”, alone or in combination. Some of the revised information is connected with previous research of our work groups and published before. More than 200 articles were analyzed for this review. Publications not included in the review are either: (1) found online only as an abstract (without access to full text); (2) not possible to translate correctly by the authors (articles not in English, German, Russian, Bulgarian, etc.); or combination of both criteria. Based on those, 43 of the results given by both databases were rejected (comprising 22% of the results).

## 3. Results and Discussion

### 3.1. Characteristics of Target Astragalus Species

The genus *Astragalus* L. is the largest in the family Fabaceae (syn. Leguminosae), with more than 3500 species [25]. *Astragalus*, excluding *Astracantha* (formerly *Astragalus* subgenus *Tragacantha*), has a world total of ca. 2500 species, of which ca. 500 are in the Americas [26]. Many of the species have conservation status “vulnerable” or “critically endangered” [27].

### 3.2. Ethnobotanical Data of Astragalus Species Used against Cancer

*Astragalus mongholicus* Bunge is the accepted name of *A. membranaceus* var. *mongholicus* (Bunge) P.G. Xiao often referred to as *A. membranaceu* [28] is a key plant in Chinese Traditional Medicine used mainly as a Qi (Chi) tonic [29,30] but also prescribed against cancer [30]. A study found that *A. membranaceus* is an ingredient in 172 of the 200 analyzed Chinese herbal formulae [31].

In Table 1 are summarized ethnobotanical data of *Astragalus* species medicinal application. Various species have been used in folk medicine as an antihypertensive, diuretic, anti-inflammatory, emollient, etc. Aerial parts, seeds, fruits, roots, or gum are utilized [32]. For instance, *A. glycyphyllos,* the herb decoction is administered in Bulgaria as an infusion in cases of abdominal pain, colic, renal inflammation, menstrual disorders, and sciatica [33]. Both roots and leaves of this plant are used as a diuretic in Italy [34]. In Turkish traditional medicine, several *Astragalus* species are used to cure throat diseases, diabetes, cardiac disorders, toothache, and abdominal pain [35,36,37,38,39,40], but also against unspecified cancer [41]. The most used herbal drug derived from the genus is Radix Astragali (roots and rhizomes of *A. mongholicus* (syn. *A. membranaceus*). The plant substance is listed in the European Pharmacopeia [42].

### 3.3. Secondary Metabolites of Astragalus Species Anticancer Properties

In recent years, progress in phytochemical studies has been made on *Astragalus* species due to their effects as immunostimulants or anticancer agents [45,47,49,50,51,52,53,54,55,56,57,58].

Many *Astragalus* species contain cycloartane saponins–astragalosides, which are cycloastragenol derivatives. Some saponins isolated from representatives of the genus are based on an oleanane skeleton [51,53]. The high intake of flavonoids is generally associated with a reduced risk of neoplasms [59,60]. Over 160 different flavonoids of 90 species of *Astragalus* have been isolated and identified as revised previously [52,53]. Polysaccharides have been shown to play a role in immune modulation. Astraglucanes have been isolated from roots and rhizomes of *A. mongholicus* (syn. *A. membranaceus*) [42] and other species. The polysaccharide fraction contains highly branched, predominantly 1,3-*β*-glucans. These products find their application as an aid in radiation antineoplastic therapy and chemotherapy, as well as in the treatment and prevention of bacterial and viral infections [61].

Different *Astragalus* extracts have been shown to increase resistance to the immunosuppressive effects of chemotherapy drugs while stimulating macrophages to produce interleukin-6 and tumor necrosis factor (TNF). Human clinical trials demonstrated a substantial increase in survival rates when extracts from *Astragalus* plants are given to cancer patients receiving chemo- or radiotherapies. They have also increased IgA, IgC, and interferon production in humans [51]. Astragaloside IV inhibited the development of non-small cell lung cancer by inhibiting the Akt/GSK-3p/p-catenin signaling pathway. It also increased the expression of Bax (a cell death marker) while decreasing the expression of Bcl-2 (anti-apoptotic protein). This demonstrates the importance of astragaloside IV as a potential antitumor agent [62].

*A. angustifolius* is an endemic Bulgarian species that has been reported to contain cyclosiversigenin (cycloastragenol), siversigenin (astragenol), and soyasapogenol B [51]. The antiproliferative activity of compounds isolated from *A. angustifolius* in cervical (HeLa), human lung (H-446), human colon (HT-29) cancer, and human monocyte lymphoma (U937) cell lines are examined [63], and only 3-*O*-[*α*-L-rhamnopyranosyl-(1→2)-*β*-D-xylopyranosyl-(1→2)-*β*-D-glucuronopyranosyl]-3β,22β,24-trihydroxyolean-12-en-29-oic acid possessed weak cytotoxicity against HeLa.

Another endemic Bulgarian plant is *A. aitosensis* which afforded 5,6-dehydro-6-desoxyastragenol [51], as shown in Table 2.

**Figure 1:**
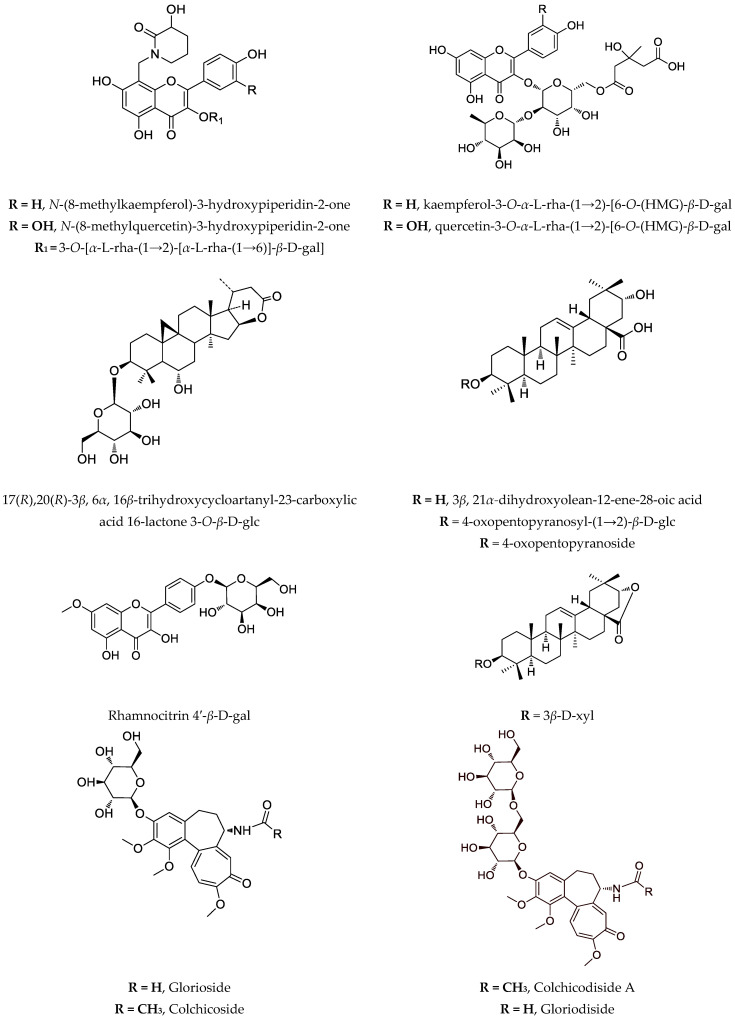

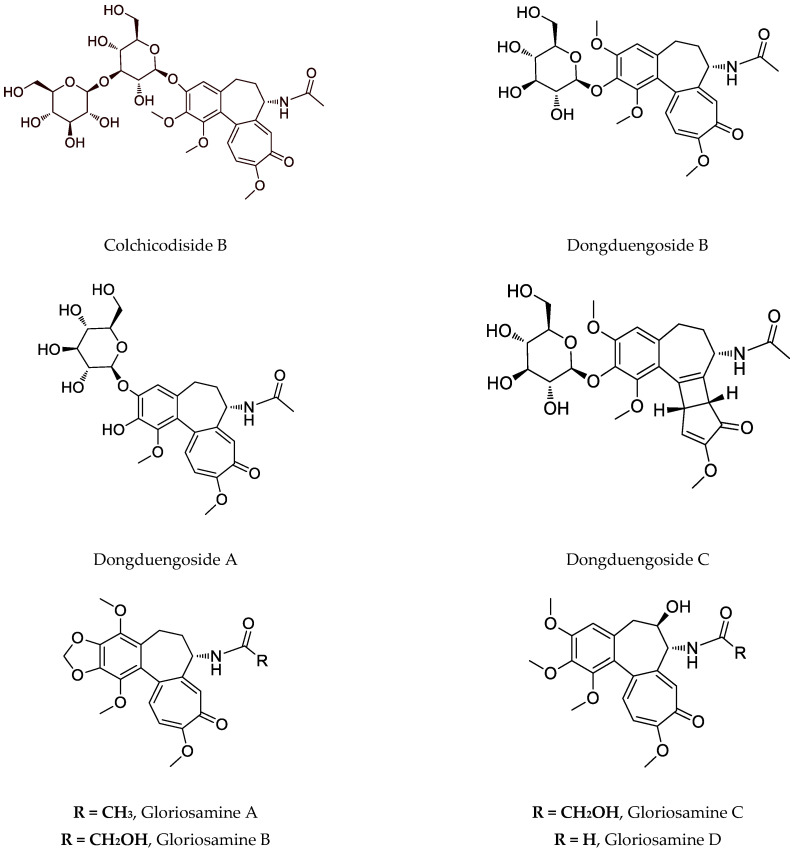
Notable compounds, isolated from *Astragalus* and *Gloriosa* species.

Recently, a novel and unusual for the genus *Astragalus* group of compounds, flavoalkaloids, is identified in *A. monspessulanus* subsp. *monspessulanus*. Before, they were known only as aglycones (Figure 1). One novel quercetin tetraglycoside and eight known flavonoids are isolated as well [53]. Also, from the aerial parts of this species, two saponins are reported [64]. Two rare flavonoids with an unusual hydroxymethylglutaric acid as a moiety: quercetin-3-*O*-*α*-L-rhamnopyranosyl-(1→2)-[6-*O*-(3-hydroxy-3-methylglutaryl)-*β*-D-galactopyranoside and kaempferol-3-*O*-*α*-L-rhamnopyranosyl-(1→2)-[6-*O*-(3-hydroxy-3-methylglutaryl)-*β*-D-galactopyranoside are isolated from the aerial parts of *A. monspessulanus* subsp. *illyricus* (Figure 1) [65].

Phytochemical investigation of *A. glycyphyllos* led to the isolation of six saponins, and their structures are partially elucidated [66,67]. After acid hydrolysis of a saponin mixture obtained from the aerial parts of the plant, soyasapogenol B and 3β,22β,24-trihidroxyolean-12-en-19-one are identified [68]. Cycloartane saponins askenoside C and F [68] and 17*(R)*, 20*(R)*-3*β*,6*α*,16*β*-trihydroxycycloartanyl-23-carboxylic acid 16-lactone 3-*O*-*β*-D-glucopyranoside are later isolated from the species [69]. Several known flavonoids, including the rare camelliaside A, are also identified [53,69]. The antineoplastic activity in vitro of the saponin-containing fractions obtained from wild-grown and cultivated *A. glycyphyllos*, respectively, were tested in a panel of human tumor cell lines of different origin and characteristics. A standard MTT-based protocol for assessing cell viability was used. Both fractions inhibited tumor cell growth in a dose-dependent manner. However, according to the calculated IC_50_ value, the fraction obtained from the in vitro shoot cultures showed relatively superior cytotoxic activity compared to that of the wild-type species in all of the screened tumor cell lines (our unpublished data). In vitro cultures of *A. glycyphyllos* could be an alternative way to produce saponins, with promising antineoplastic activity.

**Table 2:** Notable metabolites from wild and in vitro cultures of targeted species and cytotoxicity of some on a panel of malignant cells.

Plant Species	Type	Compounds Isolated	Cytotoxicity on Cell Lines (IC_50_)	References
*A. aitosensis*	callus, suspension	cycloartane saponins, sterols, flavonoids	n.d.	[51]
	aerial pars, wild grown *	5,6-dehydro-6-desoxyastragenol	n.d.	[51]
*A. angustifolius*	callus, suspension	cycloartane saponins, flavonoids	n.d.	[53]
	aerial parts, wild grown *	*β*-sitosterol, cycloastragenol, astragenol, soyasapogenol B, 3-*O*-[*α*-L-rha-(1→2)-*β*-D-xyl-(1→2)-*β*-D-glc]-3β,22β,24-trihydroxyolean-12-en-29-oic acid	n. d. HeLa (36 µM); HT-29 (50 µM)	[63] [53]
*A. asper*	aerial parts, wild grown *	saponins, flavonoids	n.d.	[53]
*A. boeticus*	callus, suspension, hairy roots	saponins, soyasapogenol B, *β*-sitosterol, flavonoids	n.d.	[70]
*A. brachycera*	hairy roots ** shoots **	cycloartane saponins, sterols	n.d.	[51]
*A. canadensis*	hairy roots	cycloartane saponins, cycloastragenol, astragenol,	n.d.	[51]
*A. centralpinus*	aerial parts, wild grown *	flavonoids	n.d.	[53]
*A. corniculatus*	aerial parts, wild grown ***	two oleanane type saponins and a corresponding lactone	Graffi tumour–in vivo, i.p., hamsters (50 mg/kg) ***; in vitro (20 µg/mL) ***	[71]
*A. edulis*	callus	quercetin, kaempferol, isorhamnetin, saponins	n.d.	[70]
*A. englerianus*	hairy roots	cycloartane saponins	n.d.	[51]
*A. falcatus*	hairy roots	cycloartane saponins	n.d.	[51]
*A. glycypyllos*	hairy roots **, callus shoots ***	cycloastragenol, astragenol, soyasapogenol B epoxycycloartanes	n.d. T-24 (125 µg/mL); CAL-29 (90 µg/mL); MJ (75 µg/mL); HUT-78 (78 µg/mL)	[51] [53] [72]
	aerial parts, wild grown ***	epoxycycloartanes	K-562 (50 µg/mL) ***; HL-60 (40 µg/mL) ***; BV-173 (70 µg/mL) ***	[73]
	aerial parts, wild grown ***	epoxycycloartanes	T-24 (168 µg/mL); CAL-29 (105 µg/mL); MJ (126 µg/mL); HUT-78 (87 µg/mL)	[72]
	aerial parts, wild grown	17(*R*),20(*R*)-3*β*,6*α*,16*β*-trihydroxycycloartanyl-23-carboxylic acid 16-lactone 3-*O*-*β*-D-glucopyranoside	T-24 (66 µg/mL); CAL-29 (52 µg/mL); MJ (52 µg/mL); HUT-78 (18 µg/mL)	[74]
*A. hamosus*	callus, suspension, hairy roots	saponins, soyasapogenol B, *β*-sitosterol, astragalin, rutin, isorhamnetin-3-O-glycoside	n.d.	[53,70]
	aerial parts, wild grown ***	saponins	HL-60 (63 µg/mL); HL-60/Dox (25 µg/mL); SKW-3 (84 µg/mL)	[75]
*A. missouriensis*	Callus **, suspension, hairy roots	isoquercitrin, quercitrin, rutin, hyperoside, saponins	n.d.	[70,71]
*A. mongholicus* (syn. *A. membranaceus*)	hairy roots **, shoots **	astragalosides, *β*-sitosterol, stigmasterol, campesterol	n.d.	[51,72]
*A. monspessulanus*	aerial parts, wild grown *	flavoalkaloids, acylated flavonoids, flavonoids	n.d.	[65]
*A. onobrychis*	aerial parts, wild grown *	flavonoids, saponins	n.d.	[76]
*A. oxyglotis*	hairy roots	cycloartane saponins	n.d.	[51]
*A. sesameus*	Shoots **	-	HL-60/Dox (87 µg/mL); SKW-3 (68 µg/mL)	
*A. spruneri*	aerial parts, wild grown *	flavonoids	n.d.	[77]
*A. sulcatus*	hairy roots	cycloartane saponins, sterols, swensonine	n.d.	[51]
*A. thracicus*	callus, suspension	saponins, flavonoids	n.d.	[53]
	aerial parts, wild grown *	saponins, flavonoids	HT-29 (52 µg/mL); HL-60 (67 µg/mL); HL-60/Dox (53 µg/mL); SKW-3 (83 µg/mL)	[53]
*A. vesicarius* ssp. *carniolicus*	callus	flavonoids	HL-60 (8.8 µg/mL) *; HL-60/Dox (11.8 µg/mL) *	[78]
	callus	5-hydroxy-7-methoxy-2′, 5′-dihydroxyisoflavone	HL-60 (38.9 µg/mL); HL-60/Dox (35.2 µg/mL)	[78]
		5, 7-dihydroxy-4′-methoxyisoflavone	HL-60 (41.4 µg/mL); HL-60/Dox (42.4 µg/mL)	[78]
		7-methoxy-5-hydroxy-4′-methoxy-2′-hydroxyisoflavone	HL-60 (64.1 µg/mL); HL-60/Dox (41.8 µg/mL)	[78]
		8-pregnyl genistein	HL-60 (36.1 µg/mL); HL-60/Dox (36.1 µg/mL)	[78]
		5,7-dihydroxy-8-pregnyl-4′-methoxy-2′-hydroxyisoflavone	HL-60 (56.3 µg/mL); HL-60/Dox (56.8 µg/mL)	[78]
		sophorophenolone	HL-60 (78.0 µg/mL); HL-60/Dox (63.0 µg/mL)	[78]
*G. superba*	seeds	colchicoside, colchicine, 3-*O*-demethylcolchicine	PANC-1, PANC02 (GS ^++^ 0.45–0.59 µg/mL) PANC02 (GS2B ^+^ 9.49 µg/mL)	[79] [80]
		glorioside, colchicodiside A, gloriodiside, colchicodiside B, colchicodiside C, dongduengoside A-C, colchicine, 2-demethilcolchicine, colchicoside and luteolin 7-*O*-*β*-D-glucopyranoside	DLA (29 µg ^#^; 21 µg ^##^)	[81] [82] [83]
	rhizomes	peptides	SW620 (n.d.)	[84]
	roots	colchicine	HT-29 (0.12 μg/mL *)	[85]
*G. rothschildiana*	aerial parts	gloriosamine A-D, colchicine, colchiciline, colchifoline and *N*-deacetyl-*N*-formylcolchicine	-	[86]

* Extract was tested; ** Extract from this culture was tested; *** Purified saponins’ mixture was tested; ^+^ GS2B, colchicine poor extract; ^++^ GS, *G. superba* total extract; ^#^ Methanolic extract of *G. superba* seeds; ^##^ AgNPs; n.d., not defined.

Three saponins are isolated from *A. corniculatus*: two of them with an aglycone 3*β*,21*α*-dihydoxyolaean-12-ene-28-oic acid, and the third–with its corresponding lactone [22,87]. A series of studies demonstrated that a purified saponin fraction containing these compounds had a protective effect against the invasiveness of bone marrow carcinoma (Graffi myeloid tumor) in hamsters. Administration of the saponin mixture increased the number, migration, and phagocytic index of peritoneal macrophages and blood polymorphonuclear leukocytes in animals with implanted tumors. Also, due to the hamster treatment with the mixture, an increased mitogenic response to phytohemagglutinin and lipopolysaccharides is observed, i.e., the saponins have an immunostimulatory effect [88,89,90].

Rhamnocitrin 4′-*β*-D-galactopyranoside and a mixture of two saponins are isolated from aerial parts of *A. hamosus* and investigated for antiproliferative activity on SKW-3 cells. Significant apoptosis-induction activity is proved for the saponin mixture compared to the flavonoid glycoside at equal concentrations. After co-administration of rhamnocitrin 4′-*β*-D-galactopyranoside, with Cisplatin and Gentamicin, there is significant protection of human kidney cells HEK-293T against the cytotoxic effects of nephrotoxic drugs [75]. The same mixture of two saponins is examined on cell lines HL-60, HL-60/Dox, SKW-3, RPMI-8226, U-266, and OPM-2 [75]. The saponins caused concentration-dependent suppression of the proliferative activity of malignantly transformed cells. These data are confirmed by an ELISA test evaluating apoptosis-specific DNA fragmentation. The significance of the transcription factor NFκB, as well as the mitochondrial protein Bcl-xL for the antitumor activity of the saponin mixture, is established. Selective cytotoxic activity of saponins in cell lines originating from breast cancer is demonstrated. The saponin mixture showed cytotoxicity concerning both cell lines and clearly demonstrated inhibitory properties against the mitochondrial anti-apoptotic protein Bcl-xL. This gives a reason to believe that unlocking the internal pathway of apoptosis by suppressing the expression of BclxL is a part of the mechanism of action of the saponins. Furthermore, the mixture of two saponins showed no cytotoxic effect on the non-malignant cell line MCF-10A, which originated from the mammary gland, suggesting that it exhibited selective malignant cell toxicity and may be the subject of further studies [91].

Data on the phytochemical content as well as the cytotoxic activity of extracts and purified fractions from wild-grown and in vitro cultivated selected *Astragalus* species on notable malignant lines is presented in Table 2. Some structures of the compounds of interest are shown in Figure 1.

All the data from the phytochemical analysis and the activity suggest that these plants are valuable as anticancer agents.

### 3.4. Biotechnology of Astragalus Species

Many in vitro cultures are established not only to increase the production of important secondary metabolites in selected *Astragalus* plants but also to preserve the endemic and/or endangered species. In general, the most investigated species both in phytochemical and biotechnological means is *A. mongholicus* (syn *A. membranaceus*) [92].

#### 3.4.1. Cell Culture

The active substances from wild and field-grown plants usually have different quality and quantity and vary depending on the environmental conditions. The diseases and the application of pesticides further reduce the quality of the plant material. In vitro plant cultures surmount these problems as environmental conditions affecting the metabolism of plants can be precisely controlled. Working with tissue cells dramatically decreases preparation time, processing, and storage costs associated with traditional plant approaches [93]. There are several advantages to producing secondary metabolites in plant cell culture compared to in vivo cultivation. Production can be more predictable, reliable, and independent from unpredictably changing climatic conditions. Isolation of the phytochemical metabolites can be more rapid and efficient than extraction from the whole plant. Interfering compounds in the wild plant can be avoided in tissue cultures. Cell cultures can produce phytochemicals in large volumes.

*Astragalus* genus possesses characteristics that make it significant for in vitro cultivation. Reducing natural supplies due to excessive collection is already present and, therefore, industrial or consumer interest. Due to their complex structures, saponins, flavonoids, and polysaccharides are still most efficiently produced by the plants. There are a lot of problems involved with this production method. Variable qualities and quantities of the plant material, plants that need to grow several years before being ready for harvesting (*Astragalus* roots), and the over-collecting of some species (*A. mongholicus,* syn. *A. membranaceus*), *A. angustifolius*, *A. missouriensis*, *A. thracicus*, *A. aitosensis,* etc.) are just a few of the problems connected with the production of these natural products. Therefore, tissue cultures might be explored as an alternative production method [7].

#### 3.4.2. Effects of Medium Composition and Growth Regulators

The flavonoid biosynthesis in cell cultures of *A. missouriensis* and *A. angustifolius* is strongly suppressed by 2,4-dichlorophenoxyacetic acid (2,4-D). The higher concentrations of 2,4-D decreased the content of flavonoids. In this respect, 1-naphthaleneacetic acid (NAA) is weaker than 2,4-D. Therefore, flavonoid production is inhibited by adding 2,4-D to the medium. In all tested concentrations of cytokinins, such as 6-benzylaminopurine (BAP) or kinetin (Kn), under the light cultivation regimen, induction of flavonoid production in cell suspension cultures is achieved. Kinetin was found to be the most effective. The sucrose levels of the medium have a tremendous impact on saponins production. The growth and the saponins production are favored by the higher concentrations of sucrose. The investigation was carried out on *A. membranaceus* hairy roots cultures (HR) with different concentrations of sucrose in the MS medium. A basic MS medium supplemented with 2% sucrose increases overall saponins yield but inhibits the growth of HR. The growth of *Astragalus*-HR is promoted by high concentration (6%), but the yield of saponins remains very low. The optimal medium for both yield and growth is supplemented with 4% sucrose [94].

#### 3.4.3. Effects of End-Product Inhibition

Because phytosterols (campesterol, stigmasterol, and *β*-sitosterol) are present in the hairy roots of *Astragalus* species, the effect on the total saponin production of these substances is examined. Since the biosynthetic route of saponins and that of phytosterols are branched at 2,3-epoxyscvalen, it may be possible to increase the production of saponins by end-product inhibition. In the experiments, contents of saponins after adding 0.2 mg/mL of *β*-sitosterol in MS liquid medium without ammonium nitrate are examined at different stages of growth. The saponin content (total saponins) in the hairy roots induced by *Agrobacterium rhizogenes* LBA 9402 reached 5.25% of dry wt on day 28 of cultivation. When *β*-sitosterol is added to the culture media of these hairy roots, astragaloside production is remarkably increased to 7.13% of dry wt and led to an increase of 36% of the total saponin content in comparison with the control. From these data, *β*-sitosterol seems to behave as an inhibitor in the biosynthetic route when the amount added is relatively large. These results prove that the hairy root cultures of *Astragalus mongholicus* can be a valuable alternative for overproducing cycloartane saponins compared with the whole plant. Using a selected high productive clone, inducing by *Agrobacterium rhizogenes* LBA 9402, optimized culture medium (MS without ammonium nitrate), and end-product inhibition, a relatively high saponin production can be achieved [94,95].

#### 3.4.4. Genetic Transformation of Astragalus Species by Agrobacterium Rhizogenes

In a specific soil, *A. rhizogenes* can induce a certain type of “hairy roots” culture, which can be maintained without phytohormones in the medium [96]. Usually, four different bacterial strains are used—TR 105, R 1601, ATCC 15834, and LBA 9402. With the same *Agrobacterium* strain, the susceptibility of *Astragalus* species to infection is highly variable. Some species of the genus (*A. membranaceus, A. mongholicus, A. monspessulanus)* have more difficulties establishing transformed roots. In some plants (*A. englerianus, A. mongholicus, A. missouriensis, A. sulcatus),* a callus is formed initially, and transformed roots subsequently emerge from it. Still, in others (*A. glycyphyllos, A. hamosus, A. boeticus*), a profusion of roots appear directly at the site of inoculation [70].

#### 3.4.5. In Vitro Production of Secondary Metabolite

Optimization of cultures and production conditions has been achieved to produce flavonoids from cell cultures of different *Astragalus* species: *A. monspessulanus*, *A. aitosensis*, *A. missouriensis*, *A. edulis*, *A. hamosus*, and *A. angustifolius* [53,65]. Flavonoids within complex plant tissues can be more difficult to separate in an intact polymeric form than cell culture-derived flavonoids. A novel vehicle for depth investigation of flavonoids individually represents the production of these compounds in uniform plant-cell culture systems.

All in vitro cultures of *A. missouriensis* and *A. angustifolius* produced flavonoids. Quercetin is the main aglycone identified in the in vitro cell suspension in both free and bound forms (as glycosides). The main flavonoid glycosides are isoquercitrine and quercitrine. Rutin and hyperoside have also been detected. The maximum total amount of flavonoids, 1.78% for *A. angustifolius* (unpublished results) and 1.34% for *A. missouriensis,* is achieved after optimization of the production medium [97].

Different types of in vitro culture lines of *A. monspessulanus* subsp. *monspessulanus*, native to Bulgaria are established, i.e., shoots, callus, and suspension. Significant differences in the flavonoid content are observed. In suspension cultures, callus, and shoot cultures, small amounts of flavonoids are quantified in comparison to wild overground parts, in which, among other flavonoids, the rare flavonoid alcesefoliside is found [98]. In vitro cultivated *A. hamosus* afforded astragalin and isoquercitrin [53]. Five isoflavonoids such as 5-hydroxy-7-methoxy-2′, 5′-dihydroxyisoflavone, 5, 7-dihydroxy-4′-methoxyisoflavone, 7-methoxy-5-hydroxy-4′-methoxy-2′-hydroxyisoflavone, 8-pregnyl genistein, 5,7-dihydroxy-8-pregnyl-4′-methoxy-2′-hydroxyisoflavone and one coumarochromone–sophorophenolone are isolated from ethylacetate fraction of in vitro callus cultures of *Astragalus vesicarius* ssp. *carniolicus*, after enzymatic hydrolysis with *β*-glucosidase and investigated for antiproliferative activity against chemosensitive human promyelocyte cell line HL-60 and its multidrug-resistant variant HL-60/Dox (Table 2). Despite the strong activity of the ethylacetate fraction, prenylated compound 8-pregnyl genistein also showed antiproliferative activity [78].

The fast growth of the biomass, as well as relatively high saponin production, can be achieved through the HR cultures of *A. mongholicus.* These produced cycloastragenol-saponins: astragalosides I-III [80,99]. Part of the saponin products (about 16–20% of the total saponin) produced by HR cultures of *Astragalus* spp. is released into the medium, essential to establish continuous saponin production. Heterogenous acid hydrolysis of the total mixture of saponins isolated from selected HR of *A. membranaceus* yielded three aglycones: astragenol, cycloastragenol, and soyasapogenol B. In vitro cultivation of *A. hamosus* and the latter phytochemical analysis of the cultures established (Table 2) revealed the presence of soyasapogenol A [51]. In vitro callus, shoot, and suspension cultures of *A. glycyphyllos* are developed when cultivated on MS, as well as using modified media (supplemented with various concentrations and combinations of plant hormones). Compared to the wild-grown species, in vitro shoot cultures accumulated double the amount of the main saponin (our unpublished data). Among them, Astragaloside (AG) IV has a tremendous interest due to its health benefits as antitumor, cardioprotective, antiviral, hepatoprotective, immunoregulatory anti-inflammatory, antidiabetic, and neuroprotective activities [51,52,81]. Due to the complicated stereochemical ring, the chemical synthesis of AG IV is difficult and commercially infeasible *A. membranaceus* hairy root cultures (AMHRCs) are developed as a biotechnology system that can supersede field-grown plants for the production of AGs [100]. However, the quantity of AG IV in AMHRCs is still low-0.02% dry weight (DW). The microbial biotransformation has been recognized to be superior to conventional chemical procedures for producing AG IV, owing to its high catalytic efficiency, inherent selectivity, low cost, and simple downstream processing [83,84]. There are many materials concerning fungus-mediated biocatalysis [83,85,86,87]. An elicitation effect of immobilized *Prunus canescens* (IPC) has been reported for in vitro cultures of plants [101]. The microorganisms have taken on greater significance in producing pharmaceuticals, chemicals, and food ingredients [89,90,102]. The fungi, especially the fungal endophytes, are a source of novel biocatalysts with numerous applications [103]. Currently, an ideal method in biotransformation procedures for producing biologically active substances represents the immobilization of microorganisms by Ca-alginate gel (CAG) [83,93,94,95]. Except through microorganisms’ biotransformation of exogenous substrates such as quercetin, kaempferol, and apigenin is achieved via suspension cultures of *Astragalus vesicarius* ssp. *carniolicus*. Respective mono-*O*-glycosylated derivatives are detected by ultra-high performance liquid chromatography-high resolution electrospray ionization mass spectrometry (UHPLC-HR-ESI-MS) analysis [104]. Suspension cultures of *A. glycyphyllos* was evaluated for possible increase in flavonoid production when treated with exogenous quercetin. Suspensions cultures, cultivated on modified G48 medium [105] supplemented with 10, 20 and 30 mg/mL quercetin achieved higher total flavonoid content (0.09, 0.10 and 0.13 mg/mg DW). In addition, biotransformation of quercetin to isoquercitrin is achieved. The highest concentration of isoquercitrin (56.73 ng/mg DW) was observed on suspensions cultures cultivated on a modified G48 medium supplemented with 20 mg/mL quercetin [106].

Many research groups have investigated the hairy root cultures of *A. mongholicus* [96,98,107] since the first report on HR *Astragalus* cultures [70]. Through the genetic transformation of *A. rhizogenes*, LBA9402 successfully induced eight *A. membranaceus* hairy root lines (I–VIII). The various AMHRCs lines showed variations in the contents of the astragalosides and isoflavonoids accumulation [100].

*A. membranaceus* field-grown roots (3-year-old) can produce 2.4 mg/g DW of total AG, while the hairy roots cultures of *A. membranaceus* can produce a higher amount (2.7 mg/g DW). The genes *RolB* and *RolC* from the plasmidic Ri-DNA of *A. rhizogenes* are responsible for the induction of AG accumulation in AMHRCs. However, for further promoting AG production in AMHRCs, there is a possibility to exploit external elicitations. There are several advantages of elicitation to induce/enhance the biosynthesis of secondary plant metabolites. Methyl jasmonate (MJ), salicylic acid (SA), and acetylsalicylic acid (ASA) are individually applied to AMHRCs to find the best elicitor for AG production. The quantity of AG increased in the range of 3.0–4.9 mg/g DW when MJ, SA, and ASA were individually fed to 34-day-old AMHRCs at 100 µM. The quantity of AG in non-treated control is 2.6 mg/g DW and 2.7 mg/g DW in ethanol-treated control. In terms of applying the three elicitors, the AG yield decreased in the following order MJ (4.9 ± 0.11 mg/g DW) > ASA (3.8 ± 0.08 mg/g DW) > SA (3.0 ± 0.15 mg/g DW). Therefore, the highest AG yield is achieved by elicitation with MJ [107].

The effect of elicitors depends on the elicitation doses [108]. Different types of UV elicitation treatments have been investigated [100,101,103,104,105,106,107,108,109,110,111,112,113,114,115,116,117,118,119,120,121,122,123,124,125,126].

### 3.5. Characteristics of Target Gloriosa Species

Genus *Gloriosa* (Liliaceae) includes 12 species and, despite its taxonomic complication, was found to be monophyletic [127]. Few of them are of commercial, pharmaceutical, or ethnobotanical interest.

#### 3.5.1. Ethnobotaical Data of *Gloriosa* Species Used against Cancer

*Gloriosa superba* is one of the plants used as an antidote against snakebite in the Southern part of Tamilnadu, India [128], and several drops of extract of this plant are rubbed onto the cuts and wounds in Rajouri and Poonch districts of Jammu and Kashmir, India [129]. It has wide application in folk medicine in tropical Africa and Asia, such as abdominal and general pain, anthelminthic and antiparasitic, leprosy, leucorrhea, mental illness, skin diseases, ulcers, etc. [130]. But also, in traditional applications in Asia and Africa, in addition to diseases such as gout, scrofula, antipyretic, anthelmintic, purgative, and antiabortive activity, anticancer use is indicated [131]. This activity is well confirmed in recent pharmacological tests against pancreatic cancer [79], colon cancer [84], and other cancer cells [132]. Due to the boom in harvesting and export trade, some populations of *G. superba* are on the edge of extinction [127].

#### 3.5.2. Secondary Metabolites of *Gloriosa* Species with Anticancer Properties

The main secondary metabolite is colchicine, which has anticancer activity but its toxicity profile is not acceptable. Several studies suggested the cytotoxic activity of semisynthetic derivatives of colchicine and thiocolchicoside; thus, the reported IC_50_ values have no relevance to naturally occurring tropolones [133,134,135,136]. Gene expression, as well as cytotoxic effects of colchicine in human gastric cancer ASG and NCI-N87 cell lines, are evaluated. It was found that only 6 ng/mL of colchicine had the desired antiproliferative effect on both lines. Interestingly, the gene regulation of those cells is affected in the same manner as the stated concentration leading to apoptosis [137]. Despite this compound, the interesting colchicinoids such as gloriosamine A-D are isolated from the aerial part of *G. rothschildiana* [86].

Nowadays, the focus on *G. superba* is also due to the presence of glycosylated colchicine derivatives, especially colchicoside, which is considered ten times less toxic than colchicine, as shown in Table 2 [79]. Recently, four novel colchicinoids named *N*-deacetyl-*N*-formyl-3-de-*O*-methylcolchicine-3-*O*-*β*-D-glucopyranoside (glorioside), 3-de-*O*-methylcolchicine-3-*O*-*β*-D-glucopyranosyl-(1→6)-3-*O*-*β*-D-glucopyranoside (colchicodiside A), *N*-deacetyl-*N*-formyl-3-de-*O*-methylcolchicine-3-*O*-*β*-D-glucopyranosyl-(1→6)-3-*O*-*β*-D-glucopyranoside (gloriodiside) and 3-de-*O*-methylcolchicine-3-*O*-*β*-D-glucopyranosyl-(1→3)-3-*O*-*β*-D-glucopyranoside (colchicodiside B) are isolated from the seeds of the species [81]. Also, from a seedless pot of Thai origin, *G. superba* was identified in novel glycosylated colchicinoids–dongduengoside A-C, and colchicine, 2-demethilcolchicine, colchicoside, and luteolin 7-*O*-*β*-D-glucopyranoside [82]. Some colchicinoids are obtained using biotechnological approaches. Riva et al. (1997) describe that *β*-1,4-galactosyltransferase catalyzes galactosylation of colchicoside, and Pišvejcová et al. (2006) describe the influence of various parameters on the activity of *β*-1,4-galactosyltransferase derived from beef milk and the optimization of the conditions leading to the preparation of galactosylate and glycosylated colchicoside derivatives [138,139].

Within a survival experiment carried out in a murine model of pancreatic adenocarcinoma induced by PANC02 cells and the semi-long-term toxicity, slightly longer survival is observed for the group treated with colchicoside rich extract (GS2B) containing 0.07% colchicine, 2.26% colchicoside and 0.46% 3-*O*-demethylcolchicine. In contrast, combinatory treatment of total seed extract of *G. superba* (GS) with gemcitabine demonstrates a significant effect on tumor growth [80]. Among 23 selected plants from the Thai/Lanna medicinal database “MANOSROI III” frequently used in the anticancer recipes, methanolic extracts of *G. superba* roots demonstrated the highest antiproliferative activity against colon cancer cell line (HT-29) (Table 2). Significantly higher, dose-dependent apoptotic morphological changes on HT-29 cancer cells at a concentration of 50 μg/mL are observed for methanol extract and the hexane, methanol-water, and n-butanol fractions obtained therefrom when compared to cisplatin and doxorubicin [85]. Methanolic extract of *G. superba* seeds is employed in forming silver nanoparticles (AgNPs) with reduced toxicity [140]. Thus, the anticancer properties of *G. superba* seeds methanolic extract and AgNPs are studied against dalton lymphoma ascites (DLA) cells (Table 2). Using the rhizome extract of *G. superba*, biomolecule-coated nanotitania catalysts were synthesized, which showed an IC_50_ value of 46.64 µg/mL MCF-7 cancer cell line compared with L929 normal cells (IC_50_ 61.81  µg/mL) [141]. In addition, in vivo study with DLA tumor-bearing mice demonstrated an increased survival rate from 20 days without treatment up to 72 days when seed extract was administered and 75 days for AgNPs treated group, respectively. Despite the undisputed activity of the phenethylisoquinoline alkaloids, major secondary metabolites, and also peptides obtained from the rhizome of *G. superba* are investigated against colon cancer [84]. In vivo study suggests that ethanol leaf extract of *G. superba* contains phytochemicals that can induce apoptosis via mitochondrial permeability transition pore opening and protect against monosodium glutamate-induced hepato-cellular injury and proliferative disorder in prostate and uterus [142]. Partially purified protein hydrolysate (30 ng/mL) significantly inhibited viability (by 40%) of SW620 human colon cancer cells and induced apoptosis by the up-regulation of p53 and down-regulation of NF-kB, considered potential targets for anticancer therapy.

#### 3.5.3. Biotechnology of *Gloriosa* Species

The medicinal interest in *G. superba* and its nonstop over-exploitation are the main reasons to apply in vitro techniques for the conservation, production, and enhancement of secondary metabolites. The species has a very low rate of seed germination as well as seed production is quite low and uneconomical. One of the problems with commercial cultivation is the poor viability of the seeds [143]. Four or five vegetative cycles are necessary for the complete reproductive phase [144]. Commercially these plants are propagated using daughter corms with a week multiplication ratio (1:1), slow and insufficient for conservation of this species [145]. Thus, in vitro cultivation is needed to conserve this taxon, otherwise we will lose it by 2020 [146]. Plant biotechnological approaches, such as in vitro mass multiplication, have been taken for the conservation, and various methods and techniques have been studied for the production and enhancement of secondary metabolites. An efficient protocol is established for in vitro micro-propagation using corm bud explant [147]. Extracts from buds inoculated at MS medium supplemented with different concentrations of 2,4-D (1.0–10.0 mg/L) and IAA (0.5–5.0 mg/L) indicated that higher concentrations of 2,4-D and IAA reduce the callus induction. The shoot initiation depends on the combination of cytokinins. Most shoots are obtained in the presence of 9.84 μM 2iP combined with 4.64 μM Kin after 21-day culturing. Sivakumar et al. (2019) developed an efficient protocol for in vitro mass propagation of *G. superba* using callus derived from non-dormant corm buds [148]. Medium supplemented with a combination of plant growth regulators such as BAP (1.5 mg/L), NAA (0.6 mg/L), and polyamine putrescine (15 mg/L) as secondary messengers in signaling pathways, induced maximum shoot buds (87.5). Within this study, optimal seed germination of 86% is also achieved when seeds are treated with 70% sulphuric acid for 2 min. Mahendran et al. (2018) initiated cell suspension cultures of *G. superba* with a callus derived from rhizomes cultivated on MS medium supplemented with 2.0 mg/L, 2,4-D, and 0.5 mg/L NAA [149].

Despite the general advantages which make the plant cell suspension cultures suitable for the production of secondary metabolites, using classical fermentation technology the opportunity to scale up for bigger manufacturing and regulatory requirements following established once for microbial and mammalian cells, plant cell suspension can serve as tools for biotransformation of foreign substrates. Biotransformation is in vitro tissue culture technique used for commercial exploitation of secondary metabolites in which chemical conversions of an exogenously supplied substance are catalyzed by microorganisms, cells, or their enzymes, including oxidation, reduction, hydroxylation, esterification, hydrolysis, methylation, glycosylation, etc. Biotransformation of colchicinoids into their 3-*O*-glucosyl derivatives using *Bacillus megaterium* is reported [150]. In vitro glycosylation of colchicine to its 3-*O*-demethylglucoside has been investigated with different modern biotechnology tools, especially using selected microbial strains such as *Bacillus aryabhattai* [151]. Only non-selective demethylation of colchicine has been obtained using a *Colchicum variegatum* culture yielding a mixture of 3-demethylcolchicine and 2-demethylcolchicine [152]. Regio-specific demethylation at the C-3 position of colchicine has been achieved when using selected bacterial microorganisms [152]. Glycosylation of exogenous thiocolchicine by plant cell suspension cultures of *Centella asiatica* resulted in monoglycosydated derivatives at C-2 and at C-3 of the aromatic ring, which is not highly selective biotransformation [153]. Zarev et al. (2018) achieved region-specifically demethylation of the C-3 methoxy group bound to the aromatic ring A of the colchicine, and subsequent glycosylation of the demethylated derivative at the same site, using plant suspension cultures of *A. vesicarius*, which normally do not produce tropolone type of alkaloids. Thus, quantitative HPLC-UV analysis showed two times fold increase in colchicoside yield of 9.35 µmol/g DW when compared to its natural source, *G. superba* seeds, 4.26 µmol/g [154].

Using modern biotechnology, Sivakumar et al. (2019) reached concentrations of colchicine (2.65 µg/mL, 3.56 µg/mL, and 5.69 µg/mL) within the methanolic extract of in vitro treated leaf samples with spermidine, spermine, and putrescine, resp. [148]. The obtained amount of colchicine is much higher when compared to the leaf samples from field-grown plants (2.41 µg/mL). Among four elicitors tested to cell suspension cultures of *G. superba*, casein hydrolysate (CH) exhibited the maximum level of colchicine production [8.290 mg/g dry weight (DW)] at 300 mg/L concentration for 15 days’ exposure, while after 30 days’ exposure salicylic acid (SA) at 27.62 mg/L concentration showed an enhanced colchicine production rate (8.149 mg/g DW), when compared to non-elicited control cultures. Treatment for 15- and 30-days with 200 and 300 mg/L AgNO_3_ resulted in thiocolchicoside content in a cell suspension culture of 4.55 mg/g DW and 1.53 mg/g DW, respectively [149]. The highest colchicine content (0.29%) in the tubers of micropropagated plantlets raised from non-dormant tuber explants is achieved when combinatory treatment of *Glomus mosseae* and *Acaulospora laevis* arbuscular mycorrhizal fungi strains is applied [155]. Also, random amplified polymorphic DNA (RAPD) and inter simple sequence repeat (ISSR) profiles of micropropagated plants of *G. superba* is compared to evaluate the possible somaclonal variations [156]. The homogeneity of the micropropagated plants is proved as amplification products showed similar banding patterns to that of non-dormant tuber explants.

## 4. Conclusions

In this review, we summarize the research efforts made in recent years concerning the secondary cytotoxic metabolites obtained from plant species belonging to the genera *Astragalus* L. and *Gloriosa* L., many of them with conservational status. Our integrative approach bonds phytochemistry with cancer therapy, offering a possible supply of valuable metabolites without contributing to the global biodiversity loss. The biotechnological production of some of the molecules in plant cell and tissue cultures obtained of the studied species preserves the wild populations of the rare ones. This way of production of the target compounds, such as isoflavonoids, saponins, etc., detected with UHPLC-HR-ESI-MS is promising and prospective as often the quantity of the desired metabolites exceeds many times that in native plants. We believe that cell cultures of species from *Astragalus* L. and *Gloriosa* L. as a source of biologically active anticancer compounds could one day play a role in large-scale processes. However, this review has only the aim of combining information to date. It could be the basis for future research on these valuable compounds and their in vitro production. Of course, a single report on the possibility of biotechnological production of a molecule is not enough for its practical production. Our findings are only a preliminary step in biotechnological production. Implications for further research are the aspects of automation, large-scale production, the stability of the reported culture itself, as well as cost-effectiveness, which are in the major part missing in most of the sources.

## Figures and Tables

**Table 1:** Ethnobotanical use of *Astragalus* species.

*Astragalus* (Incl. *Astracantha*) Species	Location	Health Disorders	Reference
*Astragalus* sp.	Turkey	Roots cooked with milk for poultice applied to abdomen	[35]
*A. amblolepis* Fisch	Turkey	Unspecified cancer	[41]
*A. abolinii* Popov	Uzbekistan	Kidney disease, hypertension, burns, demulcent	[32]
*A. americanus* (Hook.) M.E.Jones	American countries	Stomach pain and flu	[32]
*A. amherstianus* Benth.	Pakistan	Galactagogue in animals	[32]
*A. amphioxys* A.Gray	America countries	Rattlesnake bite	[32]
*A. angustifolius* Lam	Lebanon	Astringent	[32]
*A. armatus* Willd.	Algeria	Leishmaniasis, helminthiasis	[32]
*A. brachycalyx* Fisch. ex Boiss.	Iran	Laxative, febrifuge, and digestive	[32]
*A. brachycalyx* Fisch. ex Boiss	Turkey	Unspecified cancer	[41]
*A. caucasicus* Pall.	Turkey	Diabetes	[40]
*A. caucasicus* Pall.	Caucasus, Georgia	Food (tea)	[43]
*A. canadensis*	America countries	Analgesic	[32]
*A. camptoceras* Bunge	Iran	Cold	[32]
*A. cephalotes* Banks. & Sol. var. *brevicalyx* Eig.	Turkey	Diabetes, wound healing	[37]
*A. coluteoides* Willd.	Lebanon	Diabetes and jaundice	[32]
*A. chamaephaca* Freyn	Turkey	Mouth wounds	[39]
*A. crassicarpus* Nutt.	American countries	tonic, anticonvulsive and anti-headache	[32]
*A. creticus* Lam.	Pakistan	Sedative and tonic	[32]
*A. crenatus* Schult.	Iran	Kidney stone, sedative, arthrodynia, carminative	[32]
*A. cruentiflorus* Boiss.	Lebanon	Diabetes and jaundice	[32]
*A. dasyanthus* Pall.	Ukraine	Cardiovascular insufficiency and chronic nephritis	[44]
*A. effusus* Bunge	Iran	Cough	[32]
*A. fasciculifolius* Boiss.	Iran	Cough, kidney, stomach ache, chest infection, toothache	[32]
*A. fischeri* Buhse ex Fisch.	Iran	Toothache, backache, bone ache, kidney ache, bone fracture, and diabetes, and to induce abortion	[32]
*A. glaucacanthos* Fisch.	Iran	Tonic, gastric pain, headache	[32]
*A. globiflorus* Boiss.	Iran	Healing deep infectious wounds	[32]
*A. glycyphyllos* L.	Bulgaria	Abdominal pain, colic, renal inflammation, menstrual disorders, and sciatica	[33]
*A. glycyphyllos* L.	Montenegro	Increasing men’s sexual potency	[32]
*A. glycyphyllos* L.	Italy	Diuretic, kidney ailments, gout, and rheumatism.	[32]
*A. gossypinus* Fisch.	Iran	Cough	[32]
*A. grahamianus* Benth.	Pakistan	Treatment of abscesses and as an analgesic	[32]
*A. gummifer* Lab.	Turkey	Throat diseases	[36]
*A. gummifer* Lab.	Turkey	Diabetes	[38]
*A. hamosus* L.	India	Nervous system disorders; liver, kidney, and spleen infection.	[32]
*A. jolderensis* B.Fedtsch.	Iran	Typhoid and dermal problems	[32]
*A. lamarckii* Boiss.	Turkey	Ulcer	[32]
*A. longifolius* Lam.	Turkey	Cardiac disorder, diabetes	[38]
*A. microcephalus* Willd.	Turkey	Unspecified cancer	[41]
*A. microcephalus* Willd.	Iran	Asthma, strengthen hair	[32]
*A. mongholicus* Bunge	China	Qi (Chi) tonic	[29,30]
*A. mongholicus* Bunge	China	Cancer	[45,46,47]
*A. monspessulanus* L.	Italy	Diuretic	[32]
*A. mucronifolius* Boiss.	Iran	Backache	[32]
*A. noaeanus* Boiss.	Turkey	Varicosis	[32]
*A. ovinus* Boiss.	Iran	Stomachache	[32]
*A. tragalus* podolobus Boiss. & Hohen.	Iran	Abdominal pain	[32]
*A. psilocentros* Fisch.	Pakistan	Cataract and stomach problems	[32]
*A. rhizanthus* Benth.	India	Digestive disorders, leucorrhea, and urinary troubles	[32]
*A. rubrivenosus* Gontsch.	Uzbekistan	Kidney disease, hypertonic disease, burns, demulcent	[32]
*A. sarcocolla* Dymock	Jordan	Incense, pains	[32]
*A. sieversianus* Pall.	Iran	Menstrual disorders	[32]
*A. spinosus* Muschl.	Pakistan	To treat wounds	[48]
*A. thomsonianus* Benth. ex Bunge	India	Gastric troubles, swelling, and joint pains	[32]
*A. tmoleus* Boiss.	Turkey	Toothache	[32]
*A. tribulifolius* Bunge	India	Diuretic agent and to lower kidney disorders.	[32]
*A. tribuloides* Delile	Iran	Urinary infection	[32]
*A. verus* Olivier	Iran	Antiparasitic, antimycotic, and immunomodulatory activities	[32]
*A. zanskarensis Bunge*	India	Against worms	[32]

## Data Availability

Not applicable.
